# On the transport equation for probability density functions of turbulent vorticity fields

**DOI:** 10.1098/rspa.2021.0534

**Published:** 2022-01

**Authors:** Jiawei Li, Zhongmin Qian, Mingrui Zhou

**Affiliations:** ^1^ Department of Mathematical Sciences, Carnegie Mellon University, Pittsburgh, PA 15213, USA; ^2^ Mathematical Institute, University of Oxford, Oxford OX2 6GG, UK; ^3^ Oxford Suzhou Center for Advanced Research, China

**Keywords:** Navier–Stokes equation, probability density function method, turbulent flows, vorticity

## Abstract

Vorticity random fields of turbulent flows (modelled over the vorticity equation with random initial data for example) are singled out as the main dynamic variables for the description of turbulence, and the evolution equation of the probability density function (PDF) of the vorticity field has been obtained. This PDF evolution equation is a mixed type partial differential equation (PDE) of second order which depends only on the conditional mean (which is a first-order statistics) of the underlying turbulent flow. This is in contrast with Reynolds mean flow equation which relies on a quadratic statistics. The PDF PDE may provide new closure schemes based on the first-order conditional statistics, and some of them will be described in the paper. We should mention that the PDF equation is interesting by its own and is worthy of study as a PDE of second order.

## 1. Introduction

In statistical fluid mechanics (cf. [1]), the velocity U(x,t) of a turbulent flow is promoted to a random field, cf. [2], indexed by space variable $x\in R^{3}$ and time parameter t. From this point of view, the turbulence problem, if there is one, seeks for a description of the distribution of the velocity field. This distribution is rather complicated and consists of all joint distributions of the velocity across over finitely many locations and times, and therefore it is challenging to describe the distributions of turbulent flows in general. As early as the 1950s, Hopf [3] (cf. [1] too) made an ambitious attempt and derived differential equations for the distribution of a turbulent flow. Hopf’s differential equations are however infinite-dimensional and involve functional derivatives, which are therefore too difficult to extract useful information about turbulence. The dominant approach in the statistical theory of turbulence, initiated in Taylor’s seminal work [4,5], has been based on the analysis of moment structure functions and relied on the spectral method (cf. [1,6] for example). In the past decades, attention has been paid to the one-dimensional marginal distribution of the velocity field U(x,t), whose distribution is finite-dimensional. It is reasonable to assume that the distribution of U(x,t), with (x,t) fixed, has a probability density function (PDF) with respect to the Lebesgue measure on $R^{3}$. The PDF methods based on formal PDF transport equations have been developed through a series of works by Pope and other researchers (cf. [7,8]), which become powerful tools for modelling turbulent flows. The PDF, by definition, has to satisfy ‘the adjoint equation’ of the fluid dynamic equations. If the turbulent flow is an incompressible viscous fluid flow, then the PDF of the velocity must satisfy the adjoint equation of the Navier–Stokes equation. The transport equation for PDF known in the literature is still a formal adjoint equation of the Navier–Stokes equation, and therefore only few features from the transport equation may be used in modelling turbulent flows.

For incompressible fluid flows, the Navier–Stokes equation is equivalent to the vorticity equation (see (2.1) below), and therefore it is natural to consider the vorticity W=∇∧U as the main fluid dynamic variable for the study of turbulence [9]. There are good reasons why we should concentrate on the vortex motion in turbulence. The velocity of a turbulent flow is unlikely to be independent or possess conditional independence with respect to the spatial variable, while vortex motions of many turbulent flows observed in nature (such as vortex lines, vortex rings) acquire certain conditional independence, in the sense that by focusing on the motion near a fixed region, the future vortex motions evolve more or less independent of what happens in other positions. In fact there are good evidences which demonstrate that some sort of superposition property of vorticity may be maintained, although not exactly due to highly nonlinear and non-local nature of turbulence. These observations are valuable in modelling turbulent flows via the vorticity, which are already applied in vortex methods (cf. [10,11]). In this sense, PDF methods based on the vorticity are valuable.

The main contribution of the present work is the partial differential equation (PDE) for the PDF of the vorticity (called PDF PDE or PDF equation for short), which will be derived in the main body of the paper. The PDF PDE is highly nonlinear; however, the most striking aspect is that the PDF PDE for the vorticity depends only on one single first-order statistical characteristic of the turbulent flow. More precisely, we identify the main statistical characteristic needed for the PDF PDE with the conditional mean function of the increment of the vorticity given the current vorticity:

1.1 $$\mu^{i}(x,y,w,t)=E[W^{i}(y,t)-W^{i}(x,t)|W(x,t)=w],$$

for i=1,2,3, where $x,y,w\in R^{3}$ and t≥0. The PDF of the vorticity W is a solution to the PDF PDE, which is a second-order PDE where coefficients appearing in the PDF PDE depend on μ only. Besides its theoretical interest, this PDF PDE paves the way towards practical modelling the statistics of turbulent flows based on the vorticity PDF.

The paper is organized as follows. In §2, several notions and notations together with several standard assumptions about fluid dynamic random fields will be introduced, and the main result, namely the PDF PDE for vorticity, will be derived. In §§3 and 4, the theoretical foundation, based on our PDF PDE for vorticity, will be laid for the purpose of modelling various turbulent flows. In §5, we propose the most direct way of modelling PDF of the vorticity by specifying the conditional mean function $\mu^{i}$. The PDF PDE considered purely as a PDE theory is over-determined, due to its nonlinearlity in the sense that the coefficients appearing in the PDF PDE are not independent of its solutions. Therefore, care is needed to ensure that the additional constraint is satisfied. In the last section, we propose the heat flow method to model statistical quantities needed for closing the PDF PDE and obtain concrete PDF examples.

### (a) Conventions on notations

The following conventions are used throughout the paper. Firstly Einstein’s convention on summation on repeated indices through their ranges is assumed, unless otherwise specified. If A is a vector or a vector field (in the space of dimension three) dependent on some parameters, then its components are labelled with upper-script indices, i.e. $A=(A^{i})=(A^{1},A^{2},A^{3})$. The same convention applies to coordinates too. Partial derivatives of functions may be labelled with variables in sub-scripts. For example, if A(w;x,t) is a vector-valued function depending on $w,x\in R^{3}$ and t, then $\nabla_{w}A$ means the total derivatives $\left(\partial /\partial w^{i}A^{j}\right)$, $\Delta_{x}A$ means the vector $\left(\partial^{2}/\partial x^{k}\partial x^{k}A^{i}\right)$ of the Laplacians of $A^{i}$. However, as a general rule, derivatives without subscripts mean the derivatives with respect to the variable $x=(x^{i})$, unless otherwise specified for avoiding possible confusion.

The velocity vector field will be denoted by U. W=∇∧U is its vorticity so that $W^{i}=\varepsilon^{ijk}\partial /\partial x^{j}U^{k}$, where $\varepsilon^{ijk}$ are the Levi–Civita symbols.

## 2. Probability density function equation for the vorticity

In this paper, we regard the vorticity of an incompressible turbulent flow as the main dynamic variable. The goal of this section is to derive the evolution equation for the PDF of the vorticity of an incompressible turbulent flow.

### (a) Prelims and assumptions

Let U(x,t) be the velocity of an incompressible turbulent flow in $R^{3}$ with viscosity ν. Suppose there is no external force supplied to the turbulence. Being a random field though, U(x,t) satisfies the Navier–Stokes equations

$$\frac{\partial U^{i}}{\partial t}+U^{j}\frac{\partial U^{i}}{\partial x^{j}}=\nu \Delta U^{i}-\frac{\partial P}{\partial x^{i}}$$

and

$$\frac{\partial U^{j}}{\partial x^{j}}=0,$$

where i=1,2,3 and P is the pressure, subject to initial conditions which are random. The motion equations of the vorticity W=∇∧U are the vorticity equations

2.1 $$\frac{\partial W^{i}}{\partial t}+U^{j}\frac{\partial W^{i}}{\partial x^{j}}=\nu \Delta W^{i}+W^{j}\frac{\partial U^{i}}{\partial x^{j}},$$

for i=1,2,3.

We make the following technical assumptions.

First, we assume that both U(x,t) and W(x,t) have derivatives in x and t of any order, and these derivatives decay to zero at infinity sufficiently fast, so that possible boundary terms arising in applications of the Stokes’ formula have no contributions in computations below. Therefore, since U is divergence-free, ΔU=−∇∧W, according to Green’s formula

$$U^{i}(x,t)=\int_{R^{3}}\frac{1}{4\pi |x-y|}\varepsilon^{ijk}\frac{\partial }{\partial y^{j}}W^{k}(y,t)\;\text{d}y,$$

which yields the Biot–Savart law:

2.2 $$U^{i}(x,t)=-\int_{R^{3}}\varepsilon^{ijk}\frac{x^{j}-y^{j}}{4\pi |x-y{|}^{3}}W^{k}(y,t)\;dy.$$

for i=1,2,3.

Our second technical assumption is to impose certain regularity on the distribution of the vorticity. At each point x and instance t≥0, W(x,t) is a random variable defined on a probability space (Ω,F,P), taking values in $R^{3}$, and therefore its law (or called distribution) is a probability measure on $\left(R^{3},B(R^{3})\right)$. We assume that W(x,t) has a positive and smooth PDF, denoted by f(w;x,t), in the sense that

$$P(W(x,t)\in A)=\int_{A}f(w;x,t)\;dw$$

for every Borel measurable subset A. The joint distribution of W(x,t) and W(y,t) for any pair x≠y has a positive and smooth PDF, denoted by $f_{2}(w_{1},w_{2};x,y,t)$.

To state our third assumption, we first notice that

$$f_{2|1}(w;w_{1},x,y,t)=\frac{f_{2}(w_{1},w;x,y,t)}{f(w_{1};x,t)}$$

is the PDF of the conditional law of W(y,t) given $W(x,t)=w_{1}$. The conditional mean function $\mu^{i}(x,y,w,t)$ is defined by

2.3 $$\begin{array}{rl} \mu^{i}(x,y,w,t) & \;=E[W^{i}(y,t)-W^{i}(x,t)|W(x,t)=w]\\ & \;=\int_{R^{3}}(w_{1}^{i}-w^{i})f_{2|1}(w_{1};w,x,y,t)\;dw_{1}, \end{array}$$

for i=1,2,3, which will play a dominant role in the sequel.

The third technical assumption is about the regularity of the conditional average function $\mu =(\mu^{1},\mu^{2},\mu^{3})$. It is assumed that the derivatives of μ of any order exist and decay to zero sufficiently fast at infinity. Moreover, it is assumed that μ has an asymptotic expansion

2.4 $$\mu^{i}(x,y,w,t)=a_{k}^{i}(x,w,t)(y^{k}-x^{k})+b_{jk}^{i}(x,w,t)(y^{k}-x^{k})(y^{j}-x^{j})+o(|y-x{|}^{2})$$

as |y−x|→0, where $a_{k}^{i}$’s and $b_{jk}^{i}$’s are assumed to be continuous with respect to all of their arguments. We denote $b^{i}=b_{kk}^{i}$ for i=1,2,3. It is clear that

2.5 $$a_{k}^{i}(x,w,t)=E\left[\frac{\partial W^{i}}{\partial x^{k}}(x,t)|W(x,t)=w\right]={\frac{\partial }{\partial y^{k}}\mu^{i}(x,y,w,t)|}_{y=x},$$

which represents the local rate of change in the vortex motion over the turbulent region, and

2.6 $$b^{i}(x,w,t)=\frac{1}{2}E[\Delta_{x}W^{i}(x,t)|W(x,t)=w]=\frac{1}{2}\Delta_{y}\mu^{i}(x,y,w,t){|}_{y=x}.$$

In the remainder of the paper, we will work with a turbulent flow for which the three assumptions listed above are satisfied.

### (b) Probability density function equation and its derivation

In this section, we derive the main result of the paper, that is a PDE which the PDF of the vorticity must satisfy.

**Theorem 2.1.**

*Under the assumptions and notations established in §2a, suppose the PDF* f(w;x,t) *of the vorticity is smooth in* (w,x,t) *and has finite moments, that is*

$$\int_{R^{3}}|w{|}^{n}f(w;x,t)\;dw<\infty ,$$

*for every* n=1,2,… . *Then* f *satisfies the following PDE*:

2.7 $$\left(\frac{\partial }{\partial t}+\frac{\partial B^{i}}{\partial x^{i}}+B^{i}\frac{\partial }{\partial x^{i}}-\nu \Delta_{x}\right)f=\nu \frac{\partial }{\partial w^{i}}\left(\frac{\partial }{\partial x^{k}}(\;fa_{k}^{i})-2b^{i}f\right)+\frac{\partial }{\partial w^{i}}(\;fD^{i}),$$

*where* $\Delta_{x}$ *is the Laplacian with respect to the space variable* x, $a_{k}$ *and* b *are given as in* (2.5) *and* (2.6),

2.8 $$B^{i}(x,w,t)=\int_{R^{3}}\frac{1}{4\pi |y-x|}\varepsilon^{ijk}\frac{\partial }{\partial y^{j}}\mu^{k}(x,y,w,t)\;dy,$$

*and*

2.9 $$D^{i}(x,w,t)=w^{l}\int_{R^{3}}\frac{y^{l}-x^{l}}{4\pi |y-x{|}^{3}}\varepsilon^{ijk}\frac{\partial }{\partial y^{j}}\mu^{k}(x,y,w,t)\;dy.$$

**Proof.**

Let F be a smooth function on $R^{3}$ with a compact support. By the definition of PDF one has

$$\int_{R^{3}}F(w)\frac{\partial }{\partial t}f(w;x,t)\;dw=E\left[\frac{\partial }{\partial t}F(W(x,t))\right].$$

We are going to calculate the right-hand side expectation in terms of the PDF f(w;x,t) and other statistical characteristics of the turbulent flow. This will be done by using the following equation:

$$\frac{\partial }{\partial t}F(W(x,t))=\nu \Delta_{x}F(W)-\frac{\partial (U^{i}F(W))}{\partial x^{i}}+F_{j}(W)W^{i}\frac{\partial U^{j}}{\partial x^{i}}-\nu \frac{\partial F_{j}(W)}{\partial x^{k}}\frac{\partial W^{j}}{\partial x^{k}},$$

where, for simplicity, $F_{j}$ denote the partial derivatives $\partial F/\partial x^{j}$ of F, j=1,2,3. This equation follows directly from the vorticity equations. From the previous equation, we obtain that

2.10 $$\begin{array}{rl} E\left[\frac{\partial }{\partial t}F(W(x,t))\right] & \;=\nu E[\Delta_{x}F(W)]-E\left[\frac{\partial (U^{i}F(W))}{\partial x^{i}}\right]\\ & \;\;+E\left[F_{j}(W)W^{i}\frac{\partial U^{j}}{\partial x^{i}}\right]-E\left[\nu \frac{\partial F_{j}(W)}{\partial x^{k}}\frac{\partial W^{j}}{\partial x^{k}}\right]\\ & \;:=I_{1}+I_{2}+I_{3}+I_{4}. \end{array}$$

Let us calculate $I_{i}$’s on the right-hand side of equation (2.10). First, it is easy to see that by definition

2.11 $$I_{1}=\nu \Delta_{x}E[F(W)]=\int_{R^{3}}F(w)\nu \Delta_{x}f(w;x,t)\;dw.$$

For computing $I_{2}$, we shall use equation (2.2) and obtain that

$$\begin{array}{rl} I_{2} & \;=-E\left[\frac{\partial (U^{i}F(W))}{\partial x^{i}}\right]=-\frac{\partial }{\partial x^{i}}E[F(W)U^{i}]\\ & \;=-\frac{\partial }{\partial x^{i}}E\left[F(W)\varepsilon^{ijk}\int_{R^{3}}\frac{1}{4\pi |y-x|}\frac{\partial }{\partial y^{j}}W^{k}(y,t)\;dy\right]. \end{array}$$

To work out the expectation on the right-hand side, we may rewrite the partial derivative as a limit:

$$\frac{\partial }{\partial y^{j}}W^{k}(y,t)=\lim_{h\to 0}\frac{1}{h}(W^{k}(y+he^{\left(j\right)},t)-W^{k}(y,t)),$$

where $e^{\left(j\right)}$ represents the unit vector with jth component equal to 1, and the rest two components 0, so that we can rewrite $I_{2}$ as the following limit

2.12 $$I_{2}=-\frac{\partial }{\partial x^{i}}\lim_{h\to 0}\frac{1}{h}E\left[F(W)\varepsilon^{ijk}\int_{R^{3}}\frac{1}{4\pi |y-x|}(W^{k}(y+he^{\left(j\right)},t)-W^{k}(y,t))\;dy\right].$$

The expectation in this expression may be written in terms of two-point joint distributions of W as the following

$$\int_{R^{3}}F(w)\left[\int \frac{\varepsilon^{ijk}}{4\pi |y-x|}\left(\int_{R^{3}}w_{1}^{k}(\;f_{2}(w,w_{1};x,y+he^{\left(j\right)},t)-f_{2}(w,w_{1};x,y,t))\;dw_{1}\right)dy\right]dw.$$

The inner integral against the variable $w_{1}$ equals

$$f(w;x,t)(\mu (x,y+he^{\left(j\right)},w,t)-\mu (x,y,w,t)).$$

After substituting this in equation (2.12) and sending h→0, we obtain that

2.13 $$I_{2}=\int_{R^{3}}F(w)\left[-\frac{\partial }{\partial x^{i}}\left(f(w;x,t)\int_{R^{3}}\frac{\varepsilon^{ijk}}{4\pi |y-x|}\frac{\partial }{\partial y^{j}}\mu^{k}(x,y,w,t)\;dy\right)\right]dw.$$

Next we deal with $I_{3}$. Again, using (2.2), we may write

$$\frac{\partial }{\partial x^{j}}U^{i}(x,t)=\int_{R^{3}}\frac{y^{j}-x^{j}}{4\pi |y-x{|}^{3}}\varepsilon^{ilk}\frac{\partial }{\partial y^{l}}W^{k}(y,t)\;dy,$$

so that

$$\begin{array}{rl} I_{3} & \;=E\left[F_{i}(W)W^{j}\frac{\partial U^{i}}{\partial x^{j}}\right]\\ & \;=E\left[F_{i}(W)W^{j}\int_{R^{3}}\frac{y^{j}-x^{j}}{4\pi |y-x{|}^{3}}\varepsilon^{ilk}\frac{\partial }{\partial y^{l}}W^{k}(y,t)\;dy\right], \end{array}$$

which can be evaluated by using the two-point joint PDF. Indeed, we may repeat the same idea as in the computation of $I_{2}$, to obtain that

2.14 $$I_{3}=\int_{R^{3}}F(w)\frac{\partial }{\partial w^{i}}\left[-f(w;x,t)\varepsilon^{ilk}w^{j}\int_{R^{3}}\frac{y^{j}-x^{j}}{4\pi |y-x{|}^{3}}\frac{\partial }{\partial y^{l}}\mu^{k}(x,y,w,t)\;dy\right]dw.$$

Similarly, for the last term $I_{4}$, we write

$$\begin{array}{rl} I_{4} & \;=-\nu \sum_{k=1}^{3}E\left[\frac{\partial F_{i}(W)}{\partial x^{k}}\frac{\partial W^{i}}{\partial x^{k}}\right]\\ & \;=-\nu \sum_{k=1}^{3}\lim_{h\to 0}\frac{1}{h^{2}}E[(F_{i}(W(x+he^{\left(k\right)},t))-F_{i}(W(x,t)))(W^{i}(x+he^{\left(k\right)},t)-W^{i}(x,t))]\\ & \;=-\nu \sum_{k=1}^{3}\lim_{h\to 0}\frac{1}{h^{2}}\iint_{R^{3}\times R^{3}}(F_{i}(w_{2})-F_{i}(w_{1}))(w_{2}^{i}-w_{1}^{i})f_{2}(w_{1},w_{2},x,x+he^{\left(k\right)},t)\;dw_{1}dw_{2}\\ & \;=\nu \int_{R^{3}}F_{i}(w)\lim_{h\to 0}\frac{1}{h^{2}}\sum_{k=1}^{3}\{f(w;x+he^{\left(k\right)},t)\int_{R^{3}}(w_{1}^{i}-w^{i})f_{2|1}(w_{1};w,x+he^{\left(k\right)},x,t)\;dw_{1}\\ & \;\;+f(w;x,t)\int_{R^{3}}(w_{1}^{i}-w^{i})f_{2|1}(w_{1};w,x,x+he^{\left(k\right)},t)\;dw_{1}\}dw\\ & \;=\nu \int_{R^{3}}F_{i}(w)\lim_{h\to 0}\frac{1}{h^{2}}I_{4}^{h}dw, \end{array}$$

where for simplicity we have introduced the notation

$$\begin{array}{rl} I_{4}^{h} & \;:=\sum_{k=1}^{3}\{(\;f(w;x+he^{\left(k\right)},t)-f(w;x,t))\mu^{i}(x+he^{\left(k\right)},x,w,t)\\ & \;\;+f(w;x,t)(\mu^{i}(x,x+he^{\left(k\right)},w,t)+\mu^{i}(x+he^{\left(k\right)},x,w,t))\}. \end{array}$$

$I_{4}^{h}$ can be evaluated by using (2.4) when h is sufficiently small, by which we mean that

$$\mu^{i}(x,x+he^{\left(k\right)},w,t)=a_{k}^{i}(x,w,t)h+b_{kk}^{i}(x,w,t)h^{2}+o(h^{2})$$

and

$$\mu^{i}(x+he^{\left(k\right)},x,w,t)=-a_{k}^{i}(x+he^{\left(k\right)},w,t)h+b_{kk}^{i}(x+he^{\left(k\right)},w,t)h^{2}+o(h^{2}).$$

Consequently, we have

$$\begin{array}{rl} \mu^{i}(x,x+he^{\left(k\right)},w,t)+\mu^{i}(x+he^{\left(k\right)},x,w,t) & \;=-(a_{k}^{i}(x+he^{\left(k\right)},w,t)-a_{k}^{i}(x,w,t))h\\ & \;\;+(b_{kk}^{i}(x,w,t)+b_{kk}^{i}(x+he^{\left(k\right)},w,t))h^{2}+o(h^{2}), \end{array}$$

which yields that

$$\lim_{h\to 0}\frac{1}{h^{2}}I_{4}^{h}=-\frac{\partial }{\partial x^{k}}(\;fa_{k}^{i})+2f\sum_{k}b_{kk}^{i}.$$

and we may conclude that

2.15 $$\begin{array}{rl} I_{4} & \;=-\nu \int_{R^{3}}F_{i}(w)\left[\frac{\partial }{\partial x^{k}}(\;fa_{k}^{i})-2f\sum_{k}b_{kk}^{i}\right]dw\\ & \;=\int_{R^{3}}F(w)\frac{\partial }{\partial w^{i}}\left[\nu \frac{\partial }{\partial x^{k}}(\;fa_{k}^{i})-2\nu f\sum_{k}b_{kk}^{i}\right]dw. \end{array}$$

The PDF equation (2.7) now follows by substituting (2.11), (2.13)–(2.15) into (2.10).

**Remark 2.2.**

The PDE (2.7), although it appears linear, is in fact highly nonlinear, and more importantly, the coefficients a,b,B and D that define the PDE cannot be in general determined by the PDF f alone. We have seen that these functions are functionals of the conditional average function μ, hence the PDF f cannot be obtained solely through the PDF equation. However, the PDF PDE provides an appealing and new method for modelling the PDF f based on the modelling of μ alone, we will provide in this work several results on modelling the PDF f based on the PDF equation we just derived.

**Remark 2.3.**

If all coefficients a,b,B and D are considered as given, then we can pose the initial value problem for solving the PDF PDE (2.7). The good news is that then the PDF PDE is a scalar linear PDE of second order, although in general it is a mixed type of PDEs of second order on six-dimensional space. To the best knowledge of the authors of the present paper, this kind of PDEs has not been studied systemically in the existing literature.

### (c) The distribution of turbulence

As an application of the PDF PDE obtained in the previous subsection, we address a long-standing question in turbulence about the distribution of turbulent flows. It has been conjectured and verified by measurements over many years (cf. [6,12]) that the distribution of a genuine turbulent flow cannot be Gaussian, while a mathematical proof for this statement, to the best knowledge of the present authors, is not available yet. By using the PDF PDE, we are able to prove the following

**Theorem 2.4.**

*Consider an incompressible viscous* (*with viscosity* ν>0) *turbulent flow with vorticity* W(x,t). *Suppose*

(1) *the mean vorticity is constant at any instance*,
(2) {W(x,t)} *is weakly isotropic in the sense that*
  E[∇W(x,t)|W(x,t)=w]=0,
  *for all* x,w *and* t≥0, *and*
(3) *the distribution of* {W(x,t)} *is Gaussian*.

*Then the distribution of* W(x,t) *is independent of* x.

**Proof.**

Since $\left\{W(x,t):x\in R^{3}\text{ and }t\geq 0\right\}$ is a Gaussian random field, for x≠y, the joint law of W(x,t) and W(y,t) is a normal distribution on $R^{6}$ whose covariance matrix Σ(x,y) may be decomposed into blocks

$$\Sigma (x,y)=\left(\begin{array}{cc} \Sigma_{x} & \Sigma_{x,y}\\ \Sigma_{y,x} & \Sigma_{y} \end{array}\right),$$

where the dependence on t is suppressed for simplicity. The PDF of W(x,t) is given by

$$f(w;x,t)=\frac{1}{{\left(2\pi \right)}^{3/2}\sqrt{\det\Sigma_{x}}}\exp\left(-\frac{1}{2}{\left(w-m\right)}^{T}\Sigma_{x}^{-1}(w-m)\right),$$

where m denotes the mean vorticity, so that

2.16 $$\ln f(w;x,t)=-\frac{3}{2}\ln(2\pi )-\frac{1}{2}\ln\det\Sigma_{x}-\frac{1}{2}{\left(w-m\right)}^{T}\Sigma_{x}^{-1}(w-m).$$

Since under the assumption $a_{k}^{i}=0$, ψ=ln⁡f according to PDF PDE (2.7) must satisfy the following equation:

$$\frac{\partial \psi }{\partial t}=\nu \Delta_{x}\psi -B^{i}\frac{\partial \psi }{\partial x^{i}}+\nu |\nabla_{x}\psi {|}^{2}+(D^{i}-2\nu b^{i})\frac{\partial \psi }{\partial w^{i}}+\frac{\partial (D^{i}-2\nu b^{i})}{\partial w^{i}}-\nabla_{x}\cdot B$$

(which is the PDF PDE for ψ=ln⁡f in the case where $a_{k}^{i}$ vanish). Using (2.16) and by a lengthy but completely elementary computation, we deduce that

$$\begin{array}{rl} -2\frac{\partial \psi }{\partial t} & \;=\nu \Delta_{x}\ln\det\Sigma_{x}-\frac{1}{2}\nu |\nabla_{x}\ln\det\Sigma_{x}{|}^{2}+4\nu \frac{\partial b^{i}}{\partial w^{i}}\\ & \;\;-B^{i}\frac{\partial \ln\det\Sigma_{x}}{\partial x^{i}}-2\frac{\partial D^{i}}{\partial w^{i}}+2\nabla_{x}\cdot B+\nu u^{T}\Delta_{x}\Sigma_{x}^{-1}u\\ & \;\;-\nu u^{T}\left(\sum_{i}\frac{\partial \ln\det\Sigma_{x}}{\partial x^{i}}\frac{\partial \Sigma_{x}^{-1}}{\partial x^{i}}\right)u-4\nu b^{i}{\left(\Sigma_{x}^{-1}\right)}_{i\beta }u^{\beta }\\ & \;\;+2D^{i}{\left(\Sigma_{x}^{-1}\right)}_{i\beta }u^{\beta }-B^{i}u^{T}\frac{\partial \Sigma_{x}^{-1}}{\partial x^{i}}u-\frac{1}{2}\nu |u^{T}\nabla_{x}\Sigma_{x}^{-1}u{|}^{2}, \end{array}$$

where u=w−m. The last equation looks complicated, but the important point in our argument is the observation that, the left-hand side −2(∂ψ/∂t) is a quadratic function of u, while the right-hand side is a polynomial in u of degree 4, and only the last term on the right-hand side has order 4 in u. Therefore

$$\frac{1}{2}\nu |u^{T}\nabla_{x}\Sigma_{x}^{-1}u{|}^{2}=0,$$

for every u, which yields that $\nabla_{x}\Sigma_{x}^{-1}=0$, so that $\Sigma_{x}$ is independent of x. This completes the proof.

## 3. Inviscid fluid flows

There is a significant simplification in PDF PDE for an inviscid ‘turbulent’ flow, although such turbulence may not exist in nature. For inviscid fluid flows, the velocity U(x,t) satisfies the Euler equations

$$\frac{\partial }{\partial t}U^{i}+U^{j}\frac{\partial U^{i}}{\partial x^{j}}=-\nabla P\;\mathrm{and}\;\frac{\partial U^{i}}{\partial x^{i}}=0.$$

The PDF f(w;x,t) of W=∇∧U satisfies the (nonlinear) transport differential equation

3.1 $$\frac{\partial f}{\partial t}+\frac{\partial }{\partial x^{i}}(\;fB^{i})=\frac{\partial }{\partial w^{i}}(\;fD^{i}),$$

where B and D are given as in (2.8) and (2.9), respectively.

The following theorem provides a mathematical tool for modelling PDF of an inviscid turbulent flow.

**Theorem 3.1.**

*Consider PDE* (3.1) *where the data* B *and* D *are assumed as given. Assume that* B *and* D *are Lipschitz continuous in* (x,w) *uniformly in* t>0. *Suppose* f(w;x,t) *is a solution to* (3.1) *with continuous initial data* f(w;x,0), *and suppose* fD *decays to zero sufficiently fast as* |w|→∞. *Then we have the following*:

(1) f(w;x,0)≥0 *for all* $x\in R^{3}$, *then* f(w;x;t)≥0 *for all* $x\in R^{3}$ *and* t>0.
(2) *Suppose* $\int_{R^{3}}f(w;x,0)\text{d}w=1$ *for all* $x\in R^{3}$, *then* $\int_{R^{3}}f(w;x,t)\text{d}w=1$ *for all* $x\in R^{3}$ *and* t>0, *if and only if*
  3.2 $$\frac{\partial }{\partial x^{i}}\int_{R^{3}}B^{i}(x,w,t)f(w;x,t)\text{d}w=0,$$
  *for all* $x\in R^{3}$ *and* t>0.

**Proof.**

Let us define the integral curves X and Y by the following ordinary differential equation system:

$$\left\{ \begin{array}{ll} \frac{d}{ds}X^{i}(t,s)=-B^{i}(X(t,s),Y(t,s),t-s)), & X(t,0)=x,\\ \frac{d}{ds}Y^{i}(t,s)=D^{i}(X(t,s),Y(t,s),t-s)), & Y(t,0)=w. \end{array}\right.$$

Define

$$h(s)=f(Y(t,s);X(t,s),t-s)\;e^{N(w;x,s)},$$

for all s∈[0,t], where

$$N(w;x,s):=\int_{0}^{s}\left(\frac{\partial D^{i}}{\partial w^{i}}-\frac{\partial B^{i}}{\partial x^{i}}\right)(X(t,r);Y(t,r),t-r)\;dr.$$

Then clearly h(0)=f(w;x,t) and

$$h(t)=f(Y(t,t);X(t,t),0)e^{N(w;x,t)}.$$

It is clear that

$$\begin{array}{rl} \frac{d}{ds}h(s) & \;=e^{N}\frac{\partial f}{\partial w^{i}}\frac{d}{ds}Y^{i}(t,s)+e^{N}\frac{\partial f}{\partial x^{i}}\frac{d}{ds}X^{i}(t,s)-e^{N}\frac{\partial }{\partial t}f+e^{N}f\frac{\partial D^{i}}{\partial w^{i}}-e^{N}f\frac{\partial B^{i}}{\partial x^{i}}\\ & \;=e^{N}\left\{\frac{\partial (\;fD^{i})}{\partial w^{i}}-\frac{\partial (B^{i}f)}{\partial x^{i}}-\frac{\partial }{\partial t}f\right\}\\ & \;=0. \end{array}$$

Integrating with respect to the variable s over [0,t], we may conclude that

$$f(w;x,t)=f(Y(t,t);X(t,t),0)\exp\left[\int_{0}^{t}\left(\frac{\partial D^{i}}{\partial w^{i}}-\frac{\partial B^{i}}{\partial x^{i}}\right)(X(t,s);Y(t,s),t-s)\;ds\right].$$

The conservation of positivity property (1) follows immediately from this representation. To show (2) we consider the function $u(x,t)=\int_{R^{3}}f(w;x,t)\;dw$ for $x\in R^{3}$ and t≥0. If u(x,t)=1 for all x and t>0, then by integrating (3.1) over $R^{3}$ we obtain (3.2). Conversely, if (3.2) holds, by integrating (3.1) over $R^{3}$ then ∂/∂tu(x,t)=0, which yields that u(x,t)=1.

## 4. Weakly homogeneous and weakly isotropic flows

It has been pointed out in §§2 and 3, the coefficients appearing in the PDF PDE are determined by the conditional mean function μ(x,y,w,t) defined in (1.1). The significance of this statistical quantity of a turbulent flow has been demonstrated in the derivation of the PDF PDE. By definition the conditional mean describes the deviation from the local isotropicity of the turbulent flow, and therefore this statistical characteristic can be used in the classification of turbulent flows. In this section, we define weak homogeneous and weak isotropic turbulent flows, then study the PDF equation for these turbulent flows.

The homogeneity and the isotropicity can be defined in general for random fields indexed by a space variable $x\in R^{d}$, which has been introduced into the study of turbulence by Taylor [5]. The local homogeneous and local isotropic flows were introduced by Kolmogorov for formulating K41 theory (and its improved version K62 theory). According to Kolmogorov [2,13], a random field {Z(x,t)} is locally homogeneous if the conditional distribution of Z(y,t)−Z(x,s) given Z(x,s)=z is independent of (z,x,s) for any t≥s, and further it is locally isotropic if the conditional distribution of Z(y,t)−Z(x,s) are invariant under reflections and rotations. The usage of conditional mean function makes it possible to generalize these concepts to their weak versions.

We may simplify the PDF PDE for turbulent flows when the vorticity random field W(x,t) is *weakly homogeneous* in the sense that for every pair (y,x) and t>0, the conditional mean of $W^{i}(y,t)-W^{i}(x,t)$ given W(x,t)=w depends only on y−x, w and t>0 but independent of x, so that we may write

$$\mu^{i}(x,y,w,t)=\beta^{i}(y-x,w,t),$$

where $\beta^{i}(0,w,t)=0$. Then

$$a_{k}^{i}(x,w,t)=\frac{\partial \beta^{i}}{\partial x^{k}}(0,w,t),$$

and

$$b^{i}(x,w,t)=\Delta \beta^{i}(0,w,t),$$

which are still denoted by $a_{k}^{i}(w,t)$ and $b^{i}(w,t)$, though they are independent of x. Similarly

$$B^{i}(w,t)=\int_{R^{3}}\frac{1}{4\pi |y|}\varepsilon^{ijk}\frac{\partial \beta^{k}}{\partial y^{j}}(y,w,t)\;dy,$$

and

$$D^{i}(w,t)=w^{l}\int_{R^{3}}\frac{y^{l}}{4\pi |y{|}^{3}}\varepsilon^{ijk}\frac{\partial \beta^{k}}{\partial y^{j}}(y,w,t)\;dy,$$

which again are functions of (w,t) only. Therefore, the PDF PDE can be simplified to be the following mixed type PDE

$$\left(\frac{\partial }{\partial t}+B^{i}\frac{\partial }{\partial x^{i}}-\nu \Delta \right)f=\nu \frac{\partial }{\partial w^{i}}\left(a_{k}^{i}\frac{\partial f}{\partial x^{k}}\right)+\frac{\partial }{\partial w^{i}}(\;fA^{i}),$$

where $A^{i}=D^{i}-2\nu b^{i}$ and i=1,2,3.

We say that the vorticity W is *weakly isotropic*, if

E[∇W(x,t)|W(x,t)=w]=0,

for all x,w and t. By definition, W is locally isotropic in Kolmogorov’s sense, then it is weakly isotropic.

If W is weakly homogeneous and weakly isotropic, then a=0, B and A depend on (w,t) only, so for this case, the PDF PDE becomes a parabolic-transport equation

4.1 $$\left(\frac{\partial }{\partial t}+B^{i}\frac{\partial }{\partial x^{i}}-\nu \Delta \right)f=\frac{\partial }{\partial w^{i}}(\;fA^{i}).$$

After we have deduced the PDE (4.1), there is no need to assume that B and A are independent of x. Therefore, we may consider the following PDE

4.2 $$\left(\frac{\partial }{\partial t}+B^{i}\frac{\partial }{\partial x^{i}}+\frac{\partial B^{i}}{\partial x^{i}}-\nu \Delta \right)f=\frac{\partial }{\partial w^{i}}(\;fA^{i}).$$

where both B and A are functions of three variables x, w and t.

The following theorem provides the foundation for modelling weakly isotropic turbulent flows based on the vorticity PDF.

**Theorem 4.1.**

*Consider the parabolic-transport differential equation* (4.2), *where* A *and* B *are considered as given. Assume that* A(x,w,t) *and* B(x,w,t) *are Lipschitz continuous in space variable* (x,w) *uniformly in* t. *Suppose* f(w;x,t) *is a solution to* (4.2) *such that* fA *is continuous and decays to zero sufficiently fast as* |w|→∞.

(1) *If* f(w;x,0)≥0 *for all* x *and* w, *then* f(w;x,t)≥0 *for all* t≥0, x *and* w.
(2) *If* f(w;x,0) *is a PDF for every* x, *i.e. it is non-negative and* $\int_{R^{3}}f(w;x,0)\;dw=1$ *for every* x, *then so are* f(w;x,t) *for all* x *and* t>0 *if and only if*
  4.3 $$\frac{\partial }{\partial x^{i}}\int_{R^{3}}B^{i}(x,w,t)f(w;x,t)\;dw=0,$$
  *for all* x *and* t>0.
(3) *Suppose in addition* B *is independent of* w *and* $\partial B^{i}/\partial x^{i}=0$, *then if* f(w;x,0) *is a PDF for every* x, f(w;x,t) *is also a PDF for every* x *and for every* t>0.

**Proof.**

The proof is rather similar to that of theorem 3.1, so we only provide an outline. Let ${\left(M_{t}^{i}\right)}_{t\geq 0}$ (i=1,2,3) be a three-dimensional standard Brownian motion on a probability space (Ω,F,P). For every fixed t>0, we run the following stochastic differential equation (SDE) system:

$$dX^{i}(t,s)=\sqrt{2\nu }dM_{s}^{i}-B^{i}(X(t,s),Y(t,s),t-s)\;ds,\;X(t,0)=x$$

and

$$dY^{i}(t,s)=A^{i}(X(t,s),Y(t,s),t-s)\;ds,\;Y(t,0)=w,$$

which has a unique solution running up to t. Apply Itô’s formula to

$$H_{s}=f(Y(t,s);X(t,s),t-s)e^{P(s)},$$

where

$$P(s)=\int_{0}^{s}\frac{\partial A^{i}}{\partial w^{i}}(X(t,r),Y(t,r),t-r)\;dr.$$

Then

$$\begin{array}{rl} dH_{s} & \;=e^{P}\left(\frac{\partial f}{\partial w^{i}}dY_{s}^{i}+\frac{\partial f}{\partial x^{i}}dX_{s}^{i}+f\frac{\partial A^{i}}{\partial w^{i}}-\frac{\partial f}{\partial s}ds+\nu \Delta fds\right)\\ & \;=\sqrt{2\nu }e^{P}\frac{\partial f}{\partial x^{i}}dM_{s}^{i}, \end{array}$$

so that

$$H_{0}=H_{t}-\sqrt{2\nu }e^{P}\frac{\partial f}{\partial x^{i}}dM_{s}^{i}.$$

By taking expectation, one may deduce that the solution can be expressed as

$$f(w;x,t)=E\left[f(Y(t,t);X(t,t),0)\exp\left(\int_{0}^{t}\frac{\partial A^{i}}{\partial w^{i}}(X(t,s),Y(t,s),t-s)\;ds\right)\right].$$

Statements (1)–(3) can be easily shown using this expression and the argument in the proof of theorem 3.1. If B is independent of w, then, by integrating (4.1),

$$\left(\frac{\partial }{\partial t}+B^{i}\frac{\partial }{\partial x^{i}}-\nu \Delta \right)u(x,t)=0,$$

where $u(x,t)=\int_{R^{3}}f(w;x,t)\;dw$. Thus (3) follows from the uniqueness of the solution of the previous parabolic equation.

## 5. Modelling probability density function of weakly isotropic flows

In this and the next sections, several simple models based on our PDF equation for modelling vorticity distributions in turbulence are discussed. Clearly, the most straightforward way for modelling the distribution of vorticity is to assign the conditional mean function μ(x,y,w,t). The other parameters in the PDF PDE may be determined accordingly.

For practical reasons which will be clarified in our computations below, the simplest yet not trivial model for μ(x,y,w,t) should be a function of $|y-x{|}^{2}$ only and μ decays sufficiently fast at infinity. For such a model, the parameters $B^{i}$ and $D^{i}$ appearing in the PDF PDE (2.7) vanish identically. The other parameters $a_{k}^{i}$ and $b_{jk}^{i}$ are determined by the asymptotic condition as |y−x|→0. For this reason, we assign

5.1 $$\mu^{i}(x,y,w,t)=\left\{ \begin{array}{ll} -\frac{C}{6\nu }|y-x{|}^{2}, & \text{ if }|x-y|<\delta ,\\ h(|y-x{|}^{2}), & \text{ if }|x-y|\geq \delta , \end{array}\right.$$

for i=1,2,3, where δ>0 and h is a $C^{1}$-function such that h and its derivative decay sufficiently fast at infinity. C≥0 is a model parameter which should be reflecting certain physical properties of the underlying flows. The other parameters can be read out from this model easily: $a_{k}^{i}=0$, $b_{jk}^{i}=-\delta_{jk}(C/6\nu )$ and therefore $b^{i}=-(C/2\nu )$. With this model of μ, the PDF PDE is simplified to be the following parabolic-transport equation:

5.2 $$\left(\frac{\partial }{\partial t}-\nu \Delta \right)f=C\sum_{i}\frac{\partial f}{\partial w^{i}},$$

The solution to (5.2) has a nice probabilistic representation which may be read out from the proof of theorem 4.1. In fact

5.3 f(w;x,t)=E[f(Y(t,t);X(t,t),0)],

where f(w;x,0) is the PDF of the initial vorticity, and X and Y are solutions to the SDE:

$$\left\{ \begin{array}{ll} dX^{i}(t,s)=\sqrt{2\nu }dM_{s}^{i}, & X(t,0)=x,\\ dY^{i}(t,s)=Cds, & Y(t,0)=w, \end{array}\right.$$

where M is a standard three-dimensional Brownian motion. The solutions when s=t are given by

$$Y^{i}(t,t)=w^{i}+Ct$$

and

$$X(t,t)=x+\sqrt{2\nu }M_{t}.$$

Substituting these into (5.3) we obtain

5.4 $$\begin{array}{rl} f(w;x,t) & \;=E\left[f(w+Ct;x+\sqrt{2\nu }M_{t},0)\right]\\ & \;=E\left[f(w+Ct;x+\sqrt{2\nu t}\xi ,0)\right], \end{array}$$

where ξ is a random vector with the standard three-dimensional normal distribution $N(0,I_{3})$. Clearly the representation (5.4) may also be written in terms of Gaussian density

5.5 $$\begin{array}{rl} f(w;x,t) & \;=\frac{1}{{\left(4\pi \nu t\right)}^{3/2}}\int_{R^{3}}f(w+Ct,z,0)e^{-(|z-x{|}^{2}/4\nu t)}\;dz\\ & \;=\frac{1}{{\left(2\pi \right)}^{3/2}}\int_{R^{3}}f\left(w+Ct,x+\sqrt{2\nu t}z,0\right)e^{-(|z{|}^{2}/2)}\;dz. \end{array}$$

Both representations (5.4) and (5.5) may be used to evaluate f(w;x,t) by using for example Monte-Carlo scheme by sampling Gaussian random variables.

The initial PDF f(w;x,0) is determined by the initial vorticity distribution. For a turbulent flow, the initial vorticity may be written as a sum:

$$W(x,0)=\omega_{0}(x)+\varepsilon (x),$$

where $\omega_{0}(x)$ is the initial mean vorticity at the location x, and ε(x) represents a small random perturbation. It is reasonable, therefore, to assume that ε(x) has a normal distribution $N(0,\sigma {\left(x\right)}^{2}I_{3})$, where the variance σ(x) may or may not depend on the location x. However, if ε(x) is allowed to depend on the location x, then the noise ε(x) has to satisfy the divergence-free condition as well, a technical issue we will not address here in detail. It is reasonable to assume that the initial vorticity mean $\omega_{0}$ is distributed in a small region of the space. In particular $\omega_{0}$ decays to zero sufficiently fast near infinity. Under this assumption,

5.6 $$f(w;x,0)=\frac{1}{{\left(2\pi \sigma {\left(x\right)}^{2}\right)}^{3/2}}\exp\left[-\frac{|w-\omega_{0}(x){|}^{2}}{2\sigma {\left(x\right)}^{2}}\right].$$

By substituting this into the representation (5.4), we obtain

5.7 $$f(w;x,t)=E\left[\frac{1}{{\left(2\pi \sigma {\left(x+\sqrt{2\nu t}\xi \right)}^{2}\right)}^{3/2}}e^{-\left({\left|w+Ct-\omega_{0}(x+\sqrt{2\nu t}\xi )\right|}^{2}/2\sigma {\left(x+\sqrt{2\nu t}\xi \right)}^{2}\right)}\right].$$

It is easy to see that f(w;x,t) is no longer Gaussian except for special cases which we would like to discuss below.

If $\omega_{0}$ vanishes and if σ is a positive small constant, the initial distribution is homogeneous and equation (5.7) leads to a simple expression

$$f(w;x,t)=\frac{1}{{\left(2\pi \sigma^{2}\right)}^{3/2}}\exp\left(-\frac{|w+Ct{|}^{2}}{2\sigma^{2}}\right),$$

which is independent of the location x and remains a Gaussian density. The interesting feature about this model is that the variance stays as the constant $\sigma^{2}$ but new mean vorticity −Ct is created evenly after duration t. This is the case of a turbulence with a small constant random perturbation. For turbulent flows observed in nature, the initial vorticity mean $\omega_{0}$ does exist and does not vanish, and the random noise ε(x), for simplicity, may be modelled by a Gaussian random variable independent of x. Therefore when σ>0 is a small constant, we have

5.8 $$f(w;x,t)=\frac{1}{{\left(2\pi \sigma^{2}\right)}^{3/2}}E\left[e^{-\left(|w+Ct-\omega_{0}\left(x+\sqrt{2\nu t}\xi \right){|}^{2}/2\sigma^{2}\right)}\right],$$

where $\xi ∼N(0,I_{3})$.

For this case, the mean vorticity ω(x,t) at (x,t) can be evaluated. Indeed

$$\begin{array}{rl} \omega (x,t) & \;=\int_{R^{3}}\frac{w}{{\left(2\pi \sigma^{2}\right)}^{3/2}}E\left[e^{-\left(|w+Ct-\omega_{0}\left(x+\sqrt{2\nu t}\xi \right){|}^{2}/2\sigma^{2}\right)}\right]dw\\ & \;=E\left[\int_{R^{3}}\frac{w}{{\left(2\pi \sigma^{2}\right)}^{3/2}}e^{-(|w+Ct-\omega_{0}\left(x+\sqrt{2\nu t}\xi \right){|}^{2}/2\sigma^{2})}dw\right]\\ & \;=E\left[\omega_{0}\left(x+\sqrt{2\nu t}\xi \right)\right]-Ct, \end{array}$$

and therefore

5.9 $$\omega^{i}(x,t)=\int_{R^{3}}\frac{\omega_{0}^{i}(y)}{{\left(4\pi \nu t\right)}^{3/2}}e^{-(|y-x{|}^{2}/4\nu t)}dy-Ct.$$

This equality shows that the vorticity mean ω(x,t) under this simple model is independent of the noise parameter $\sigma^{2}$, and ω(x,t) evolves according to the heat type equation

$$\frac{\partial }{\partial t}\omega (x,t)-C=\nu \Delta \omega (x,t)\;\mathrm{and}\;\omega (x,0)=\omega_{0}(x),$$

which is a rather crude approximation to the mean vorticity equation.

## 6. Heat flow method

In this section, we propose another model for the PDF of the vorticity, based on the heat flow method, in which the conditional mean function μ is generated by a random field R(x,τ). The random field R(x,τ) evolves according to the heat flow

6.1 $$\left(\frac{\partial }{\partial \tau }-\nu \Delta \right)R^{i}(x,\tau )=0,$$

where $R(x,\tau )=(R^{1}(x,\tau ),R^{2}(x,\tau ),R^{3}(x,\tau ))$. The initial value $R(x,0)=\xi (x)=(\xi^{i}(x))$ is a centred Gaussian noise white in space in the sense that the covariance of $\xi^{i}$ at two locations u and v in $R^{3}$ is given by

6.2 $$E[\xi^{i}(u)\xi^{i}(v)]=\delta (u-v).$$

Also, we assume that $\xi^{i}$’s are i.i.d. random variables. For simplicity, we assume that $R^{i}$ is centred here, but our argument can definitely be generalized to the case when it is non-centred.

The solution to equation (6.1) is given by

6.3 $$R^{i}(x,\tau )=\int_{R^{3}}\frac{1}{{\left(4\pi \nu \tau \right)}^{3/2}}e^{-(|x-y{|}^{2}/4\nu \tau )}\xi^{i}(y)\;\text{d}y,$$

so that R(x,τ) is a centred Gaussian random field with its covariance

$$\begin{array}{rl} \sigma_{\tau }(x,y) & \;=\int_{R^{3}}\int_{R^{3}}\frac{1}{{\left(4\pi \nu \tau \right)}^{3}}e^{-(|x-u{|}^{2}+|y-v{|}^{2}/4\nu \tau )}E[\xi^{i}(u)\xi^{i}(v)]\;\text{d}u\text{d}v\\ & \;=\int_{R^{3}}\int_{R^{3}}\frac{1}{{\left(4\pi \nu \tau \right)}^{3}}e^{-(|x-u{|}^{2}+|y-v{|}^{2}/4\nu \tau )}\delta (u-v)\;\text{d}u\text{d}v,\\ & \;=e^{-(|x-y{|}^{2}/8\nu \tau )}\int_{R^{3}}\frac{1}{{\left(4\pi \nu \tau \right)}^{3}}e^{-(|u-(\left(x+y\right)/2){|}^{2}/2\nu \tau )}\;\text{d}u\\ & \;=\frac{1}{8{\left(2\pi \nu \tau \right)}^{3/2}}e^{-(|x-y{|}^{2}/8\nu \tau )}. \end{array}$$

It follows that the conditional distribution of R(y,τ) given R(x,τ)=w has a normal distribution with mean $e^{-(|x-y{|}^{2}/8\nu \tau )}w$ and covariance matrix

$$\frac{1}{8{\left(2\pi \nu \tau \right)}^{3/2}}(1-e^{-(|x-y{|}^{2}/4\nu \tau )})I_{3}.$$

Therefore, the conditional mean of R(y,τ)−R(x,τ) given R(x,τ)=w can be easily found to be

$$-(1-e^{-(|x-y{|}^{2}/8\nu \tau )})w,$$

which will be our μ(x,y,w,t) with τ=φ(t) a reparametrization as part of the model. For simplicity let us consider the power law model, that is

6.4 $$\tau =\lambda_{1}{\left(t+\lambda_{2}\right)}^{\alpha },$$

where $\lambda_{1}$ and $\lambda_{2}$ are two positive numbers which then become our model parameters. Since

$$\mu (x,y,w,t)=-\frac{|x-y{|}^{2}}{8\nu \tau }w+o(|x-y{|}^{2})w,$$

therefore $a_{k}^{i}=0$ and $b_{jk}^{i}=-\delta_{jk}(1/8\nu \tau )w^{i}$. In particular,

6.5 $$b^{i}(w,t)=-\frac{3w^{i}}{8\nu \lambda_{1}{\left(t+\lambda_{2}\right)}^{\alpha }},$$

for i=1,2,3. Since the conditional mean function μ depends only on $|x-y{|}^{2}$ and decays exponentially fast at infinity, $B^{i}=0$ and $D^{i}=0$, which implies that

6.6 $$A^{i}(w,t)=\frac{3w^{i}}{4\lambda_{1}{\left(t+\lambda_{2}\right)}^{\alpha }}.$$

Thus the PDF PDE with these parameters is reduced to the simple parabolic-transport equation

$$\left(\frac{\partial }{\partial t}-\nu \Delta_{x}\right)f=\frac{\partial }{\partial w^{i}}(A^{i}f).$$

The divergence of A is given by

$$\frac{\partial A^{i}}{\partial w^{i}}(x,w,t)=\frac{9}{4\lambda_{1}{\left(t+\lambda_{2}\right)}^{\alpha }}.$$

If α≠1 but α>0, by using the Feynman–Kac formula we have

6.7 $$f(w;x,t)=\theta {\left(\alpha ,t\right)}^{3}E[f(Y(t,t),X(t,t),0)],$$

where we have introduced

6.8 $$\theta (\alpha ,t)=\exp\left[\frac{3{\left((t+\lambda_{2}\right)}^{1-\alpha }-\lambda_{2}^{1-\alpha })}{4\lambda_{1}(1-\alpha )}\right],$$

for simplicity, and X and Y are solutions to SDE

$$\left\{ \begin{array}{ll} dX^{i}(t,s)=\sqrt{2\nu }dM_{s}^{i}, & X(t,0)=x,\\ dY^{i}(t,s)=\frac{3Y^{i}(t,s)}{4\lambda_{1}{\left(t-s+\lambda_{2}\right)}^{\alpha }}\;ds, & Y(t,0)=w. \end{array}\right.$$

If α=1 then

6.9 $$f(w;x,t)=\lambda {\left(t\right)}^{3}E[f(Y(t,t),X(t,t),0)],$$

where

6.10 $$\lambda (t)={\left(\frac{t+\lambda_{2}}{\lambda_{2}}\right)}^{3/4\lambda_{1}}.$$

The previous SDEs have explicit solutions:

$$X(t,t)=x+\sqrt{2\nu }M_{t}$$

and

Y(t,t)=wθ(α,t),

if α≠1, and

Y(t,t)=λ(t)w,

if α=1.

Therefore, if α>0 and α≠1, by plugging these explicit solutions X and Y into (6.7) we have

6.11 $$\begin{array}{rl} f(w;x,t) & \;=\theta {\left(\alpha ,t\right)}^{3}E\left[f\left(w\theta (\alpha ,t),x+\sqrt{2\nu }M_{t},0\right)\right]\\ & \;=\theta {\left(\alpha ,t\right)}^{3}E\left[f\left(w\theta (\alpha ,t),x+\sqrt{2\nu t}\xi ,0\right)\right] \end{array}$$

where $\xi ∼N(0,I_{3})$.

As in the previous section, the initial vorticity is assumed to be of the form

W(x,0)=ω(x)+ε(x),

where ε(x) has a normal distribution $N(0,\sigma^{2})$ and ω(x) is the mean vorticity at x, so that

$$f(w;x,0)=\frac{1}{{\left(2\pi \sigma^{2}\right)}^{3/2}}\exp\left[-\frac{|w-\omega (x){|}^{2}}{2\sigma^{2}}\right]$$

and therefore, according to (6.11),

$$f(w;x,t)=\theta {\left(\alpha ,t\right)}^{3}E\left[\frac{1}{{\left(2\pi \sigma {\left(x+\sqrt{2\nu t}\xi \right)}^{2}\right)}^{3/2}}e^{-\left({\left|w\theta (\alpha ,t)-\omega (x+\sqrt{2\nu t}\xi )\right|}^{2}/2\sigma {\left(x+\sqrt{2\nu t}\xi \right)}^{2}\right)}\right],$$

where $\xi ∼N(0,I_{3})$. Hence f(w;x,t) is no longer Gaussian density unless σ(x) is independent of x and ω=0 identically.

Let us discuss a special case where ω=0 identically and σ>0 is constant. Then

$$f(w;x,t)=\frac{\theta {\left(\alpha ,t\right)}^{3}}{{\left(2\pi \sigma^{2}\right)}^{3/2}}\exp\left[-\frac{|w\theta (\alpha ,t){|}^{2}}{2\sigma^{2}}\right],$$

is a Gaussian density with mean 0 and variance $\rho^{2}I_{3}$, where

6.12 $$\rho^{2}(x,t)=\frac{1}{\sqrt{\theta (\alpha ,t)}}\sigma^{2}.$$

If σ>0 is a constant and ω does not vanish identically, then f(w;x,t) is not Gaussian, but has a nice representation:

6.13 $$f(w;x,t)=\frac{\theta {\left(\alpha ,t\right)}^{3}}{{\left(2\pi \sigma^{2}\right)}^{3/2}}E\left[e^{-\left({\left|w\theta (\alpha ,t)-\omega (x+\sqrt{2\nu t}\xi )\right|}^{2}/2\sigma^{2}\right)}\right],$$

where $\xi ∼N(0,I_{3})$, and θ(α,t) is defined by (6.8).

A similar discussion applies to the model where α=1, which is certainly interesting too. For this model, the PDF f(w;x,t) is given by

6.14 $$f(w;x,t)=\lambda {\left(t\right)}^{3}E\left[f\left(w\lambda (t),x+\sqrt{2\nu t}\xi ,0\right)\right],$$

where $\xi ∼N(0,I_{3})$ and λ(t) is defined by (6.10).

Suppose again the initial vorticity

W(x,0)=ω(x)+ε(x),

where ε(x) has a normal distribution $N(0,\sigma^{2}(x))$ and ω(x) is the mean. Then

6.15 $$f(w;x,t)=E\left[\frac{\lambda {\left(t\right)}^{3}}{{\left(2\pi \sigma {\left(x+\sqrt{2\nu t}\xi \right)}^{2}\right)}^{3/2}}e^{-\left({\left|w\lambda (t)-\omega \left(x+\sqrt{2\nu t}\xi \right)\right|}^{2}/2\sigma {\left(x+\sqrt{2\nu t}\xi \right)}^{2}\right)}\right],$$

which is not Gaussian except for the following special case.

If σ(x)=σ>0 is a constant and ω(x)=0, then

$$f(w;x,t)=\frac{\lambda {\left(t\right)}^{3}}{{\left(2\pi \sigma^{2}\right)}^{3/2}}e^{-(|w\lambda (t){|}^{2}/2\sigma^{2})},$$

is Gaussian with mean zero and variance $\rho^{2}I_{3}$, where

$$\rho^{2}(x,t)=\sigma^{2}{\left(\frac{\lambda_{2}}{t+\lambda_{2}}\right)}^{3/2\lambda_{1}}.$$

The most interesting case for the purpose of modelling turbulent flows is the model where σ>0 is a small parameter and ω is not zero, so that

$$f(w;x,t)=\frac{\lambda {\left(t\right)}^{3}}{{\left(2\pi \sigma^{2}\right)}^{3/2}}E\left[e^{-({\left|w\lambda (t)-\omega \left(x+\sqrt{2\nu t}\xi \right)\right|}^{2}/2\sigma^{2})}\right],$$

where $\xi ∼N(0,I_{3})$. Although it is no longer Gaussian, some of its features can be extracted by doing Monte–Carlo simulations.

We may conclude that this model provides some nice features of the propagation of the vorticity which may be helpful for the understanding of the energy dissipation in turbulence, and we will explore this in a separate work.

## Data accessibility

This article has no additional data.
